# Rapid appearance quality of rice based on machine vision and convolutional neural network research on automatic detection system

**DOI:** 10.3389/fpls.2023.1190591

**Published:** 2023-08-17

**Authors:** Yangfan He, Baojiang Fan, Lei Sun, Xiaofei Fan, Jun Zhang, Yuchao Li, Xuesong Suo

**Affiliations:** College of Mechanical and Electrical Engineering, Hebei Agricultural University, Baoding, China

**Keywords:** appearance quality of rice, convolutional neural network, machine vision technology, rapid detection, image processing techniques

## Abstract

**Introduction:**

In the process of rice production and storage, there are many defects in the traditional detection methods of rice appearance quality, but using modern high-precision instruments to detect the appearance quality of rice has gradually developed into a new research trend at home and abroad with the development of agricultural artificial intelligence.

**Methods:**

In this study, we independently designed a fast automatic rice appearance quality detection system based on machine vision technology by introducing convolutional neural network and image processing technology. In this study, NIR and RGB images were generated into five-channel image data by superposition function, and image are preprocessed by combining the Watershed algorithm with the Otus adaptive threshold function. Different grains in the samples were labeled and put in the convolutional neural network for training. The rice grains were classified and the phenotype data were analyzed by selecting the optimal training model to realize the detection of rice appearance quality.

**Results and discussion:**

The experimental results showed that the resolution of the system could reach 92.3%. In the detection process, the system designed with this method not only reduces the subjectivity problems caused by different detection environments, visual fatigue caused large sample size and the inspector’s personal factors, but also significantly improves the detection time and accuracy, which further enhances the detection efficiency of rice appearance quality, and has positive significance for the development of the rice industry.

## 1 Introduction

Among the most important food crops in the world, rice occupies an unshakable position. Most Chinese take rice as their main food, with an average annual consumption of about 180 million tons (Meng et al., 2019). With the influence of various factors, such as the quantity production of hybrid rice, improvement of residents’ quality of life, and diversification of market development in our country, the rice industry has abandoned the previous model of blindly pursuing high yield and begun to develop in the direction of improving rice quality (Liu et al., 2017). In the process of rice production and storage, the traditional detection methods of rice appearance quality are mainly executed through artificial autonomous identification, that is to say, it is identified by the human eye combined with the intuitive analysis of the reference substance. The method of manual detection and evaluation are not only time consuming and labor intensive, but the results are also basically affected by the subjectivity of professional inspectors. The subjectivity of inspectors and the difference in judging standards caused by different regions make the rapid, automatic and accurate detection of rice appearance quality become the development trend of the industry.

With the development of artificial intelligence in agriculture, the rice quality test with the help of modern and high-precision instrument becomes a new trend at home and abroad. The rice quality inspection technologies that have been developed in recent years include texture analysis technology, near-infrared spectroscopy technology, machine vision technology, scanning electron microscopy technology, intelligent sensory technology, and image processing technology have been developed. In response to the problems of labor intensity, high cost and unstable detection accuracy of manual inspection, Ruan (2021) designed a machine vision-based rice inspection system using image processing technology, which realized the counting of the number of rice grains and the identification of incomplete grains. However, the system needs to improve the discrimination rate of the number of grains and has some problems such as single detection result and the lack of practical application value. Furthermore, Xing and Luo (2021) proposed a data fusion processing algorithm based on image processing to achieve the segmentation of rice samples and background, maximizing the elimination of noise and improving the accuracy of the subsequent detection function. Beyond that, six kinds of rice were selected as the test samples, and the system achieved the detection of broken rice rate, rice species detection, and crack detection for random rice samples, which is better than the traditional manual detection methods. However, the system has a single sample size and lacks the ability to be widely applied. Furthermore, Leethanapanich et al. (2016) observed the morphology of translucent, chalky and treated rice using scanning electron microscopy at an accelerating voltage of 10kv, and analyzed the effect of soaking and drying conditions on rice chalkiness. As revealed by the result drying at a temperature higher than the glass transition temperature of starch also promoted starch granule rearrangement, further decreasing the chalkiness of the rice. Although scanning electron microscopy can capture finer and clearer images of rice compared with industrial cameras, it is more costly and unsuitable for the development of rice inspection systems Zhang et al. (2007) designed a set of electronic nose system suitable for the detection of rice mildew, which tested the rice with different mildew levels. As shown by the experimental results, the system has a high analytical accuracy for the detection of rice mold degree. The system relies on gas sensors, such as ethanol vapor, ammonia and carbon monoxide, to achieve the analysis of rice mold degree. However, the system is a complex process to implement and is highly dependent on the detection environment. Rice texture can be assessed by trained experts through sensory testing, but this method has some disadvantages such as high labor intensity and subjective bias. In order to address this drawback, Liu et al. (2020) used a texture analyzer equipped with a multi-squeezed cell probe to monitor changes in force and evaluated the taste quality of rice by simulating the process of rice in the mouth. The texture analysis technique was mainly used to detect the steaming taste quality of rice, yet this technique lacks intuitiveness relative to appearance detection. Besides, Siriphollakul et al. (2017) used near-infrared spectroscopy for non-destructive determination of edible quality of KDML105 rice through single grain transmission method. Partial least squares regression analysis was used for accurate prediction of straight-chain starch content in raw rice with small prediction error. At the same time, Sampaio et al. (2018) used a combination of near infrared spectroscopy and chemometrics to allow accurate quantification of straight-chain starch content in rice varieties. The partial least squares analysis and other methods Partial Least Squares Regression (PLSR) were also validated, and the results showed satisfactory results for all estimates. Compared with the traditional laboratory methods, this method has the advantages of simple operation, low cost, high speed, low chemical waste, and non-destructive detection, which is suitable for “online” analysis. The combination of NIR spectroscopy and chemometrics is a simple, rapid and reliable method for quantifying straight-chain starch.

Based on the current technology and the previous research, the study has cited image processing technology, near-infrared technology, and machine vision technology for inspecting the rice appearance quality. Although many intelligent inspection methods have emerged as alternatives to manual labor in rice inspection technology, the most widely used and rapidly developing rice appearance quality inspection technology is based on the image processing technology (Image Processing) (Ma et al., 2018). Specifically, Wu et al. (2021) described the application and development prospects of digital image processing technology in fruit and vegetable grading inspection. In addition, Zhang et al. (2021) designed an image sensing technology-based appearance inspection and grading method to analyze the size, weight, and blemish points of baby vegetables. Besides, Du et al. (2018) put forward a non-destructive grading method for garlic seed quality based on the image processing for the problems of easy damage of garlic seeds and high machine cost consumption in garlic seed grading process. With certain application value in garlic seed quality grade screening, it achieves garlic seed quality screening and meets the requirement of normal working in real time.

The current detection model mainly relies on the three-channel value of RGB images, which has the defects of variability and instability. Therefore, near infrared technology (NIR) has been proposed, and near infrared images can capture a smoother seed surface. Simply speaking, the principle is that infrared light has a longer wavelength, which is less likely to cause scattering and refraction on the surface and is more penetrating. Thus, it is not blocked by the pigments on the epidermis, which could better reflect the information of the epidermis itself. Additionally, Mei (2020) designed a multispectral fusion network arithmetic based on convolutional neural network for color and NIR images, which can not only effectively fuse color and NIR images but also generate high-quality fusion images containing texture information of NIR images and color information of color images. This image is characterized by low generation noise, complete details, and good color. Tang et al. (2020) proposed a convolutional neural network-based NIR and low-illumination visible light fusion algorithm, which has an excellent performance in subjective effects and various objective indicators, advancing the application of deep learning to practical NIR and low-illumination visible light fusion problems.

The discussions above still provide insufficient reasons to support the intelligent detection of rice appearance quality. However, the development of machine vision technology has promoted the intelligent and automatic development of the current food crop appearance detection technology. For the detection technology of rice and other grains, Zhou (2016) uses machine vision technology and makes comparison with manual detection. The former has advantages of high accuracy, high efficiency and simple repetitive operation. Adopting machine vision technology for grain grading to replace traditional manual detection is a new trend to improve detection efficiency in the future. Since the adoption of machine vision technology can effectively avoid errors brought by some external factors and many subjective factors occurring in manual detection, Chen et al. (2018) proposed that the grain quality detection based on machine vision technology has basically had the advantages of non-destructive, rapid and high recognition rate. The application of machine vision technology in the detection of food quality has realized the non-destructive detection of food quality, which has a considerable prospect in the detection of food quality, and will become a research hotspot at home and abroad in future (Zhao, 2021).

Considering the shortcomings and advantages of previous work, this study independently designed a hardware device for rice appearance quality detection equipped with a software operating system by taking machine vision technology as the leading technology and combining image processing technology and near infrared technology. As for the method, it proposed for the first time to generate five-channel image data by stacking NIR and RGB images, by which the accuracy and stability of the model are improved. The images are preprocessed by combining the Watershed algorithm with the Otus adaptive threshold function, which can accurately segment the 20g (800-1000) rice grains specified in the national standard. Different grains were labeled and put in the convolutional neural network for training. The optimal training model was selected to classify the rice and analyze its phenotypic data. It was proved that the resolution of the model could reach 92.3%.

GB1354-2018 was used as the basis for rice classification in this paper. Chinese National standard GB1354-2018 has stipulated various terms and definitions, quality requirements, classification, inspection rules and inspection methods of rice. Among them, imperfect grain is characterized by diseased spot, mildew and unripe, and perfect grain is characterized by full grain, no disease spot and no mildew.

In the process of rice appearance quality detection, this system not only reduces the subjectivity problems caused by different detection environments, visual fatigue caused by large sample size and inspector’s personal factors, but also significantly improves the detection time and accuracy, which enhances the detection efficiency of rice appearance quality and grade determination. Thereby, it has realized the automatic, scientific and accurate detection of rice appearance quality, and has become a good substitute for the traditional detection method.

## 2 System introduction

### 2.1 The overall structure of the system

**Figure 1:**
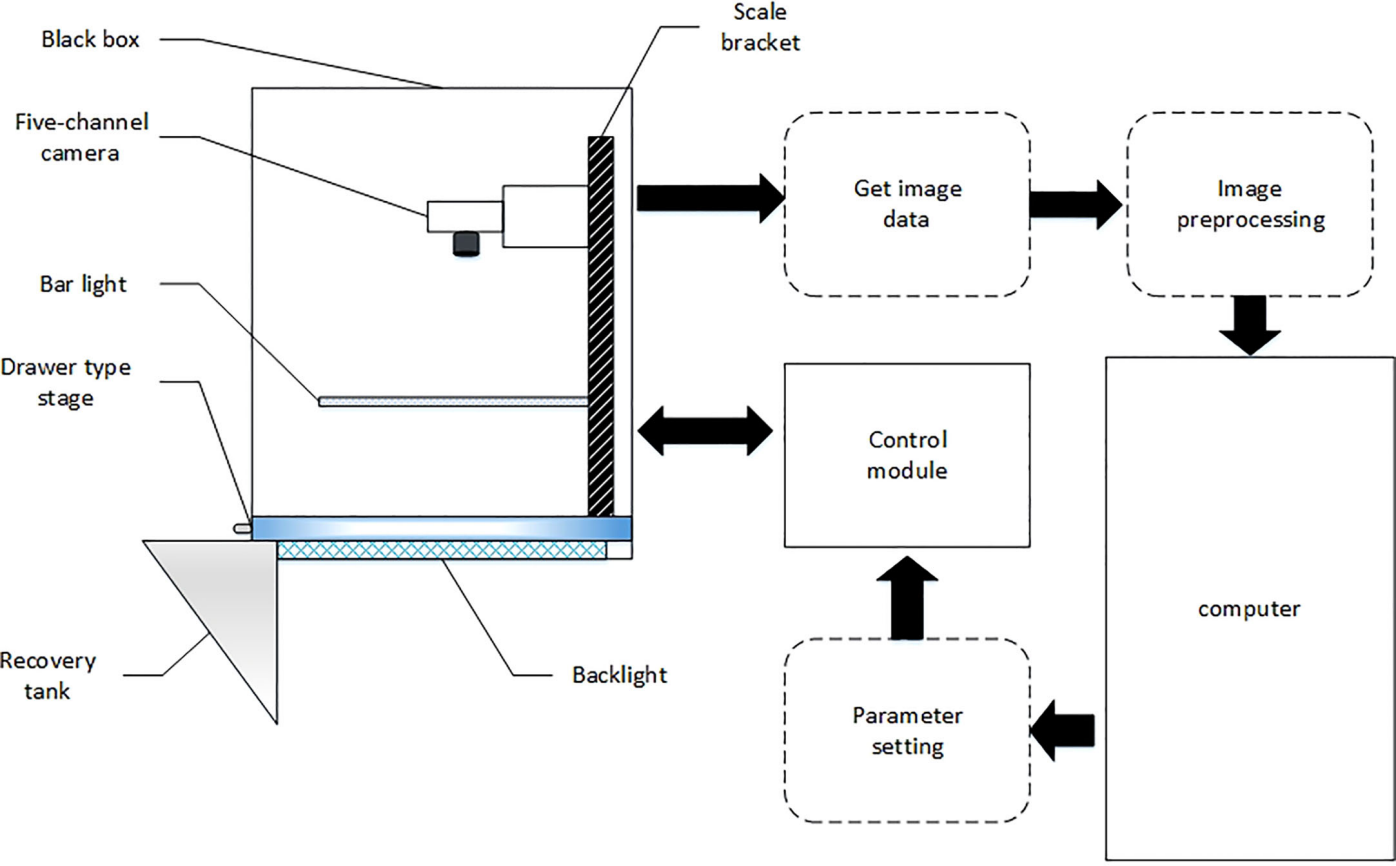
The overall structure of the system.

**Figure 2:**
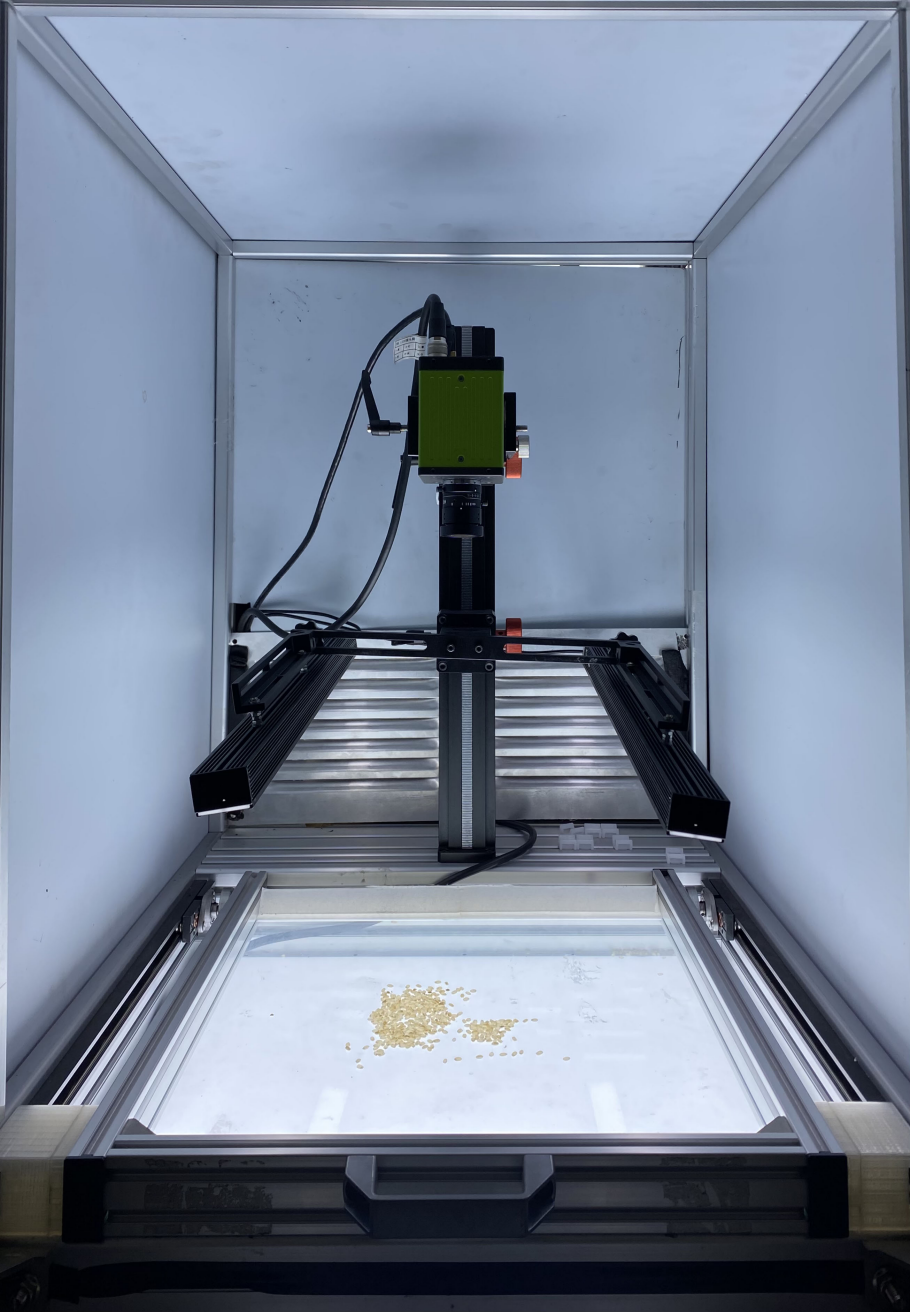
System physical.

The rice appearance quality inspection system designed independently in this study includes the dark box, strip light source, five-channel camera, drawer type carrier table, scale holder, backlight board, control module, and computer. This system has advantages of high efficiency and high precision in comparison to the current stage of manual inspection in rice appearance quality inspection. It excludes the subjectivity of manual work, and improves the inspection efficiency in time. Its general framework is shown in **Figure 1**, and the physical diagram of the system is displayed in **Figure 2**.

In this study, a five-channel camera (R, G, B, NIR_1_, NIR_2_), a global shutter 3CMOS camera under the JAI brand, model FS-3200T-10GE-NNC, was used to transmit image data through the GIGE interface. The working principle is that each of the rear end of the spectral prism is equipped with an independent light-sensitive chip to simultaneously capture color visible light images and black-and-white NIR images, color visible light images and black-and-white NIR images through dual-channel output. Moreover, the metal frame of the dark box is made of aluminum alloy, which not only ensures the simplicity of the frame but also has the durability to avoid the damage caused to the dark box or the frame later in the process of moving. The metal frame of the dark box is made of aluminum alloy. The light barrier of the dark box is made of acrylic board, which adopts opaque design to ensure that the imaging environment of the dark room is not disturbed by the external light, and has the advantages of non-breakable, light weight and high durability. In order to ensure the uniformity of lighting, this article designs a strip light source that integrate visible and near-infrared light. The visible light source is an LED light source. Near-infrared light is an electromagnetic wave located between visible light and mid-infrared light, and the wavelength is generally in the range of 780-2526 nm (Cheng et al., 2022). The detection is completed based on the characteristic of different hydrogen-containing groups in the tested sample having different reflectance values towards near-infrared light (Zhang et al., 2018). The NIR light source uses NIR light in the wavelength range of 700-1000 nm, and the images of rice in this wavelength range can well meet the experimental requirements. The strip light source is designed symmetrically on the left and right side to avoid uneven scattering of light. In addition, there is an LED backlight board with an area of 400×460cm at the bottom of the dark box to ensure the high quality of the sample images. This paper designs a drawer-type glass loading table. The drawer type has the advantages of easy operation and fast speed, which lays the foundation for rapid sample detection and replacement. The glass material has good light transmission and can effectively utilize the role of a light source and improve the quality of the sample images. In the process of drawer pulling, the inertial force can avoid the phenomenon of superposition of rice seeds. If there is a partial superposition of seeds, the phenomenon can be eliminated by shaking the drawer.

### 2.2 System working processes

In this paper, convolutional neural networks (CNNs) has been invoked to quickly and automatically detect the appearance quality of seeds using machine vision-based image processing techniques (Xiao, 2020; Wang et al., 2021). The working steps of this system are as follows: checking the integrity of the rice appearance quality inspection equipment and connecting the power supply; starting the computer, entering the account number in the login interface, and moving to the camera parameter setting interface and finally to the image acquisition and analysis interface; taking 500-1000 seeds and laying them flat on the drawer-type carrier table; turning on the strip light source and LED backlight board; conducting image acquisition and processing; manipulating the said five-channel camera to capture images and the said image acquisition and analysis interface to transform and display said image, clicking the named data analysis button to complete the said image of rice quality analysis results; completing the image acquisition, pulling out the drawer-type carrier table along the slide, tilting the rice into the rice recycling tank; putting the new rice in the drawer-type carrier table for a new test, and completing a new set of rice testing operation.

## 3 Experimental methods

### 3.1 Image pre-processing

Image contains noise or impurities when converting color images to binary images due to interference from equipment or external environment. Therefore, in this paper, we performed image noise and impurity removal on the obtained binary images (Cui et al., 2021), which in turn could make rice counting and phenotype data extraction more accurate.

There are multiple methods to remove noise points in image processing techniques. At present, the more common methods to remove noise points include Gaussian filtering, median filtering, mean filtering, etc. Binary image noise points mainly appear in the white spots in the background part other than the main part of the rice. For this feature, this study adopted the Gaussian filtering method to remove the noise points (Liang and Ma, 2017). The impurity removal function was added to Gaussian filtering to remove the impurities in the binary image.

### 3.2 Image segmentation

Comprehensively considering the requirements of practical application and detection standards, the image acquired by the system designed in this study contains 20g (800-1000 grains) of rice grains. However, using single grain rice images as input for training in convolutional neural network models yields better result, so it is necessary to conduct image segmentation processing on the pre-processed image. The connected region was labeled by Roundness indicators, and the connected areas of single rice grain and the connected areas of adhered rice were divided. Then the segmentation algorithm combining watershed algorithm and Otus adaptive threshold algorithm was applied to accurately segment the grains in adhered areas.

**Figure 3:**
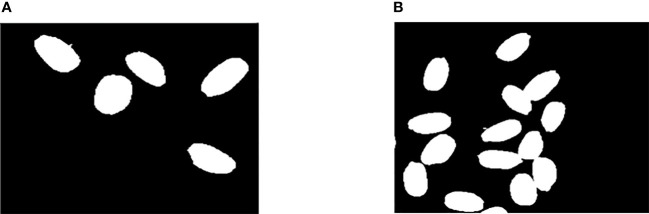
Rice Connected Area. **(A)** Connected area of single grain rice. **(B)** Conglutinated rice connected area.

After removing the noise and impurities, we found that the rice was in two states in the rice image, in which one was the single grain rice state and the other was the adhesive rice state (see **Figure 3**). The difficulty is how to deal with the adhesive rice state.

In this study, the single grain rice and the adhesive rice were first segmented effectively. The method of connected area detection is selected for effective division (Ren et al., 2021). We label the connected region in the preprocessed binary image, the labeling results were detected by the Roundness index, according to which the connected area of single rice grains and adhesive rice connected area were divided (Liu et al., 2019). The Roundness index has been defined in Equation.


(1) $$Roundness=\frac{4\pi A}{S^{2}}$$

Where, A is the area of the connected region; S is the perimeter of the connected region. The marked connected region of adhesive rice was segmented. At present, the commonly used segmentation methods include notch detection method, DPC+K-means algorithm and watershed algorithm. According to the characteristics of the image and in view of the computational volume, the Watershed algorithm is chosen in this paper (Mo, 2020). The calculation process of Watershed is an iterative labeling process. Firstly, the gray level of each pixel is sorted from low to high. Then, in the process of achieving flooding from low to high, the first-in-first-out (FIFO) structure is used to judge and label each local minimal value in the influence domain of h-order height. The watershed indicates the maximum value point of input image. Therefore, in order to obtain the edge information of the image, the gradient image is usually taken as the input image. The gradient image is calculated by Sobel operator (Han et al., 2020). namely,


(2) g(x,y)=grad(f(x,y))={[f(x,y)−f(x−1,y)]2[f(x,y)−f(x,y−1)]2}0.5

where f(x,y) denotes the original image, and grad(f(x,y)) denotes the gradient operation.

Although the watershed algorithm has a good respond to weak edge, it can produce the phenomenon of excessive segmentation. This phenomenon can be eliminated by threshold processing of gradient image. In the process of threshold processing, it is particularly critical to obtain an appropriate threshold value, which has a great impact on the quality of the final segmentation image. Therefore, Otus adaptive threshold algorithm (Luo and Zhang, 2018; Yao et al., 2020) is added, which can well avoid the disadvantage of manually adjusting the threshold value.

### 3.3 Image superposition

**Figure 4:**
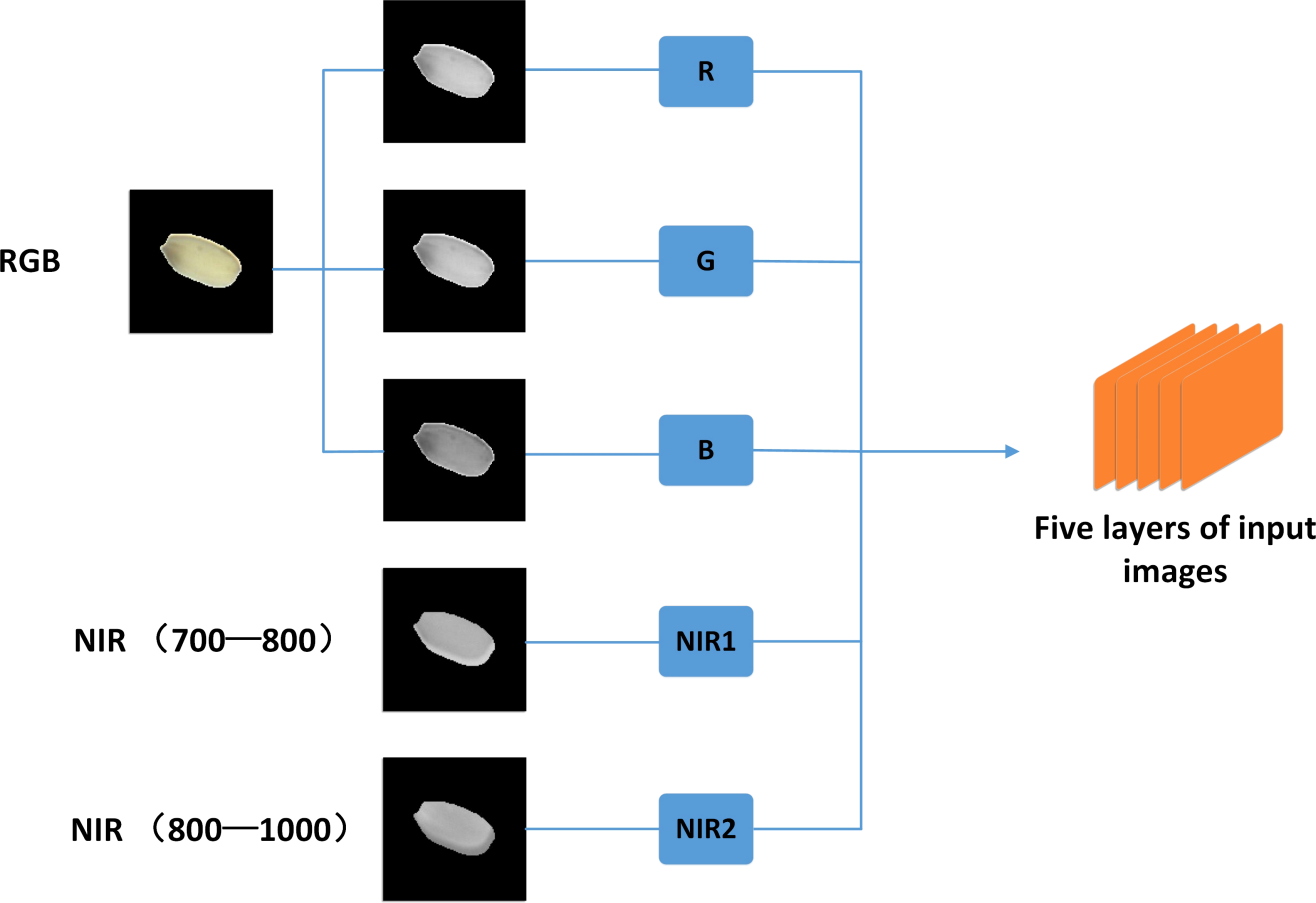
Five-layer input image.

In order to improve the stability and accuracy of the training model, this paper has used a five-channel camera, the working principle of this camera is to use prism splitting technology to split the original image onto three sensors, and can obtain three images, RGB, NIR_1_ and NIR_2_, respectively. In order to enable the three separate images to be treated as one image as input in the model training, the RGB images are firstly separated into R, G, and B channel images. Then, they are uniformly superimposed with NIR_1_ and NIR_2_ into a five-channel (RGB+NIR+NIR) image with a pixel size of 100 × 100 and the number of layers is five layers (see **Figure 4**).

### 3.4 Convolutional neural network analysis model

In this study, a convolutional neural network model was used to detection the goodness of rice, because in the current processing and transportation process, it is time-consuming and subjective to manually discriminate the goodness of rice. Therefore, an alternative to manual judgment is urgently needed, deep learning is adopted to train the model for rice (Wang et al., 2021). Meanwhile, the model with better accuracy for rice determination model is selected for the rice quality detection system after comparison. Below is the training process for different detection models.

#### 3.4.1 Analysis of VGG19 model

**Figure 5:**
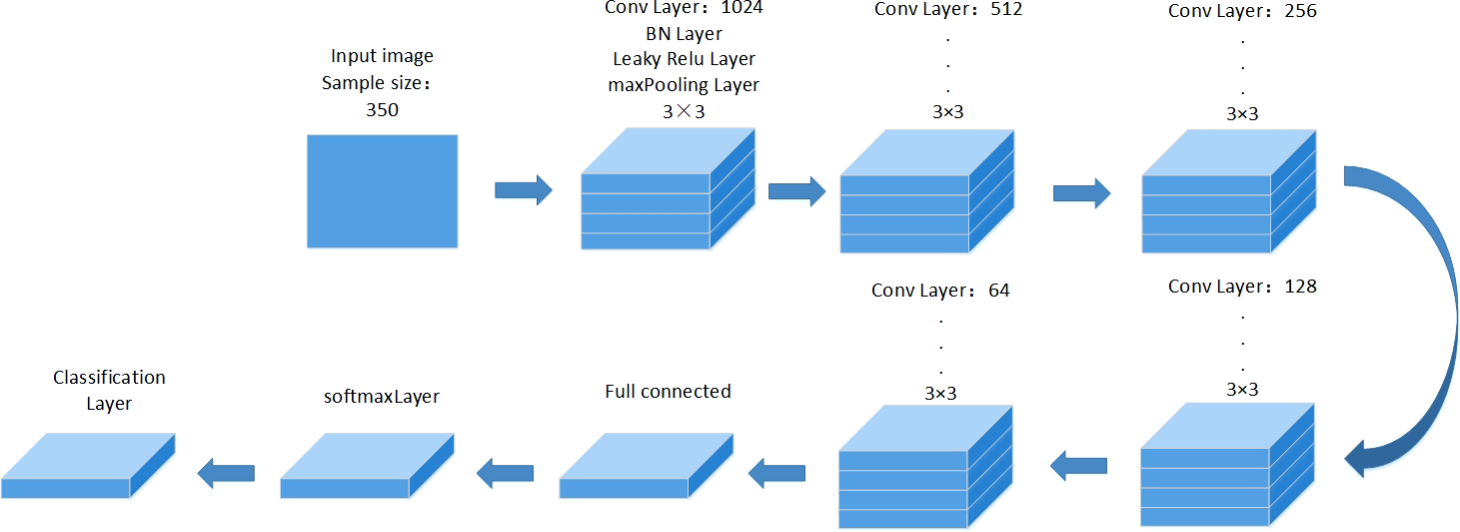
VGG19 network model architecture.

The model is a CNN model with changes based on the VGG19 network. The network model has a total of 24 layers, which are divided into one input layer, five convolutional layers, five activation layers, five pooling layers, one fully connected layer, five batch normalization layers, softmax function, and one output layer (Ishengoma et al., 2021; Wan et al., 2021). In this experiment, the input layer of the VGG19 model is set as a five-channel rice image with a pixel size of 100 × 100. Both the maximum pooling layer and the softmax function are applied to the network. The advantage of the maximum pooling layer is that it minimizes overfitting. The maximum number of training arguments is set to 100 rounds and the learning rate is 0.001. The network architecture is shown in **Figure 5**. A total of 2464 images from the pre-processed five-channel images are used, with 2100 as the training set and 364 as the test set. In addition, the VGG19 model is trained to do perfect and imperfect classification training on rice.

#### 3.4.2 Analysis of Resnet50 model

Resnet, also known as residual neural network, adds the idea of residual learning to the traditional convolutional neural network so as to avoid the phenomenon of learning degradation occurs as the number of layers of the network model deepens (He et al., 2016). In essence, the idea of residual learning can be understood as a block, which can be defined by Equation (3). Where Y denotes the output, F(X,{Wi}) denotes the residual part, and x denotes the sample.


(3) Y=F(X,{Wi})+x

**Figure 6:**
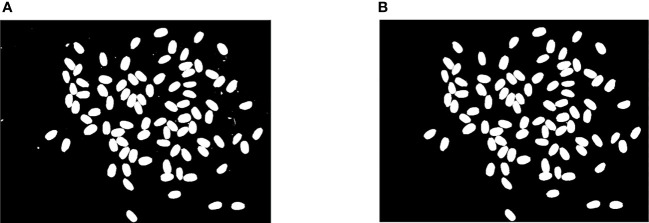
Comparison of binary image denoising and impurity removal. **(A)** Binary graph with noise or impurities. **(B)** Binary graph of impurity and noise removal.

**Figure 7:**
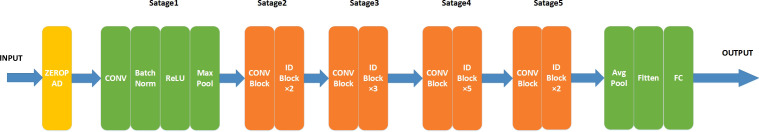
Resnet50 network architecture.

Resnet50 consists of 49 convolutional layers and 1 fully connected layer (Wang et al., 2019). The structure is shown in **Figure 6**. Where, ID BLOCK x2 in the second to fifth stages represents two residual blocks that do not change the size, CONV BLOCK represents residual blocks with added scales, and each residual block contains three convolutional layers. Therefore, there are 1 + 3 × (3 + 4 + 6 + 3) = 49 convolutional layers, in which CONV represents the convolutional layer for the convolution operation, Batch Norm represents the regularization process, Relu represents the activation function, and MAX POOL and Avg POOL represent the maximum pooling layer and the average pooling layer. As shown in **Figure 7**, ResNet50 is stacked by several residual blocks, and the deep network can be trained with these residual blocks. A total of 2464 images from the pre-processed five-channel images are used, with 2100 as the training set and 364 as the test set. Besides, the set up Resnet50 model is trained to do perfect and imperfect classification training on rice.

## 4 Experimental results

### 4.1 Image processing results

On the basis of Gaussian filtering, bwareaopen function was added to remove impurities in the binary image, and the effect was shown in **Figure 6**. The method works well in the practical application. By processing the binary image, the noise points and impurities in the image acquisition process can be effectively removed to make sure that the subsequent image segmentation and data processing are more accurate.

**Figure 8:**
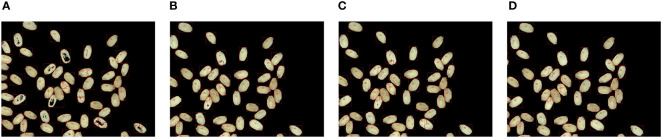
Segmentation effect comparison chart. **(A)** Manual threshold segmentation (threshold 190). **(B)** Manual threshold segmentation (threshold 200). **(C)** Manual threshold segmentation (threshold 210). **(D)** Adaptive threshold segmentation.

The image can be divided into single rice connected region and adhesive rice connected region by the connected region marker. The binary image is classified into single rice connected region and adhesive rice connected region for better segmentation of sticky rice. Then, the combination of adaptive threshold function and watershed algorithm is applied to the adhesive region rice so as to achieve better segmentation effect. The segmentation contrast effect is presented in **Figure 8**, which can reduce the computational effort of the segmentation algorithm while segmenting the target accurately. Besides, the adaptive threshold function can well avoid the tedious process brought by hand-adjusted thresholding, which not only solves the over-segmentation phenomenon of the watershed algorithm, but also provides convenience for the adjustment of the threshold.

**Figure 8** shows the optimization results of watershed combined with adaptive threshold segmentation algorithm. **Figures 8A–C** are all obtained by manual threshold value, and rice grains in the figure all have over-segmentation to varying degrees, while **Figure 8D** is the result of watershed combined with adaptive threshold segmentation algorithm. In the figure, the effect of rice grain segmentation is good without over-segmentation.

### 4.2 Model testing results

#### 4.2.1 VGG19 model testing

**Figure 9:**
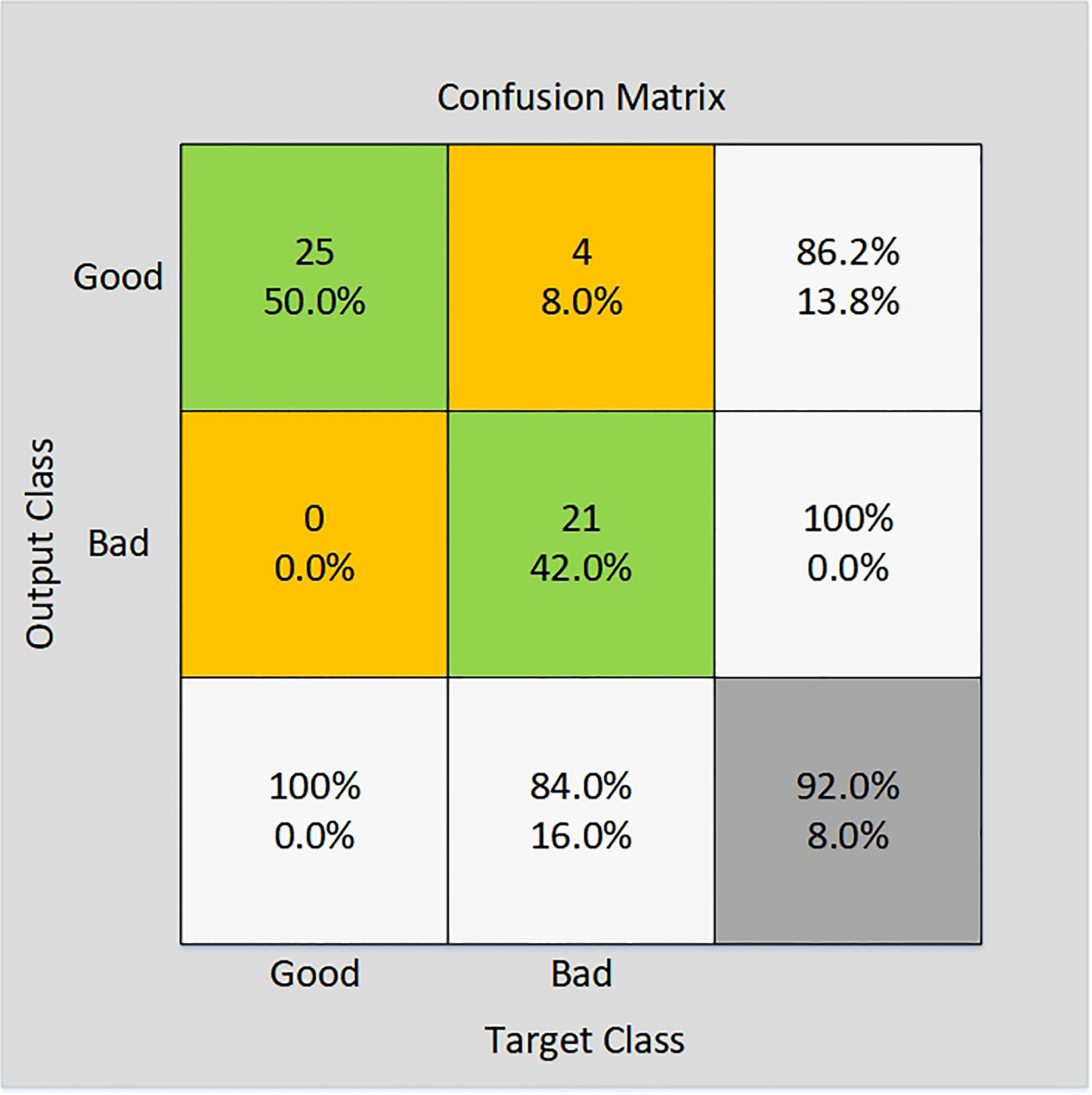
Confusion matrix of VGG19 validation set.

In this study, five-channel images of rice were used as input to VGG19 model, and the VGG19 model so as to classify rice. 25 perfect grain images and 25 imperfect grain images were collected and processed as verification sets. The size of five-channels rice grain image is 100×100 pixels. In order to verify that the final accuracy is not too high or too low due to overfitting or underfitting, VGG19 is used to train the five-channel image of rice for several times. The number of iterations in each training is different. By comparing the training accuracy of several times, it is found that the accuracy deviation is small. The final training accuracy rate is the average of multiple training accuracy rates. The accuracy of verification set results is shown through the confusion matrix, as shown in **Figure 9**, and the classification accuracy of the validation set of this model is displayed in **Table 1**.

**Table 1:** Classification accuracy of the validation set of the VGG19 model.

Appearance quality	Imperfect rice (number of grains/accuracy rate)	Perfect rice (number of grains/accuracy rate)	Classification accuracy	Verification of the number of rice grains (incomplete grains/perfect grains)
Classification accuracy	21/86.2%	25/100%	92%	25/25

As shown by the test results, 25 perfect grain images and 25 imperfect grain images are used as verification sets. The results showed that the classification accuracy for rice quality VGG19 model achieved was 92%. 21 out of 25 imperfect grains were identified, indicating an accuracy of 86.2%. All the 25 images of the perfect grains were identified, indicating 100% accuracy. After the adjustment of the model, the accuracy of the model has been significantly improved, and the stability has also been enhanced. The model meets the actual production needs, has strong practical application value, and has far-reaching influence on the rice appearance quality detection technology.

#### 4.2.2 Resnet50 model test

**Figure 10:**
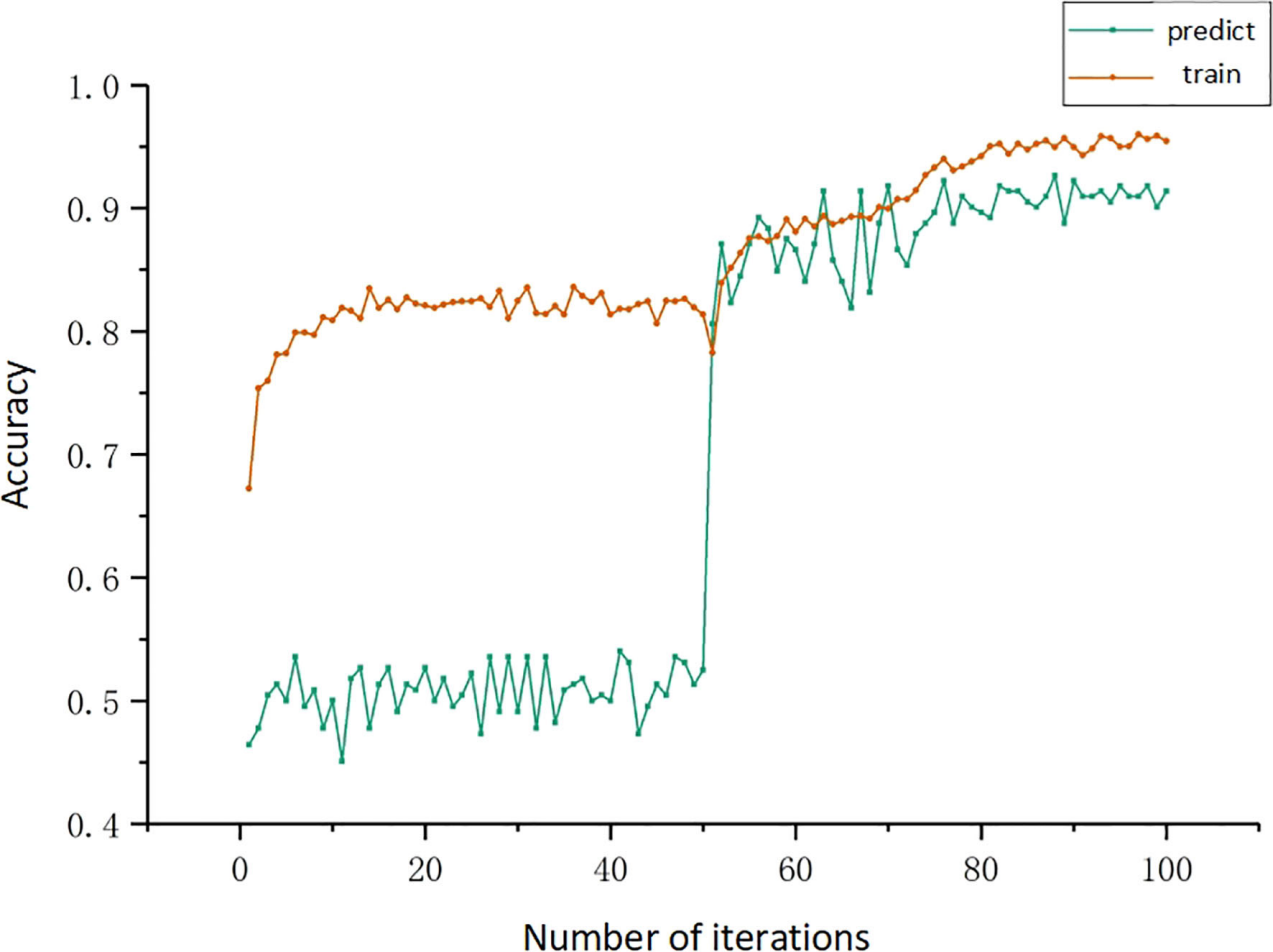
Resnet50 network training prediction curve.

A total of 2464 pre-processed five-channel images were put into the Resnet50 model for training. The iteration times were 100 times. The rice grains were put into the Resnet50 model for training of classifying perfect and imperfect grains. Similarly, the prediction of perfection and imperfection is carried out, and a total of 200 pieces of images of perfect grains and 200 pieces of images of imperfect grains are selected as the verification set. The training and verification process are shown in **Figure 10**, and the accuracy of the model is shown in **Table 2**.

**Table 2:** Classification accuracy of prediction set of Resnet50 model.

Appearance quality	Imperfect rice (number of grains/accuracy rate)	Perfect rice (number of grains/accuracy rate)	Classification accuracy	Predict the number of rice grains (incomplete grains/perfect grains)
Classification accuracy	182/91%	183/91.5%	91.3%	200/200

**Figure 11:**
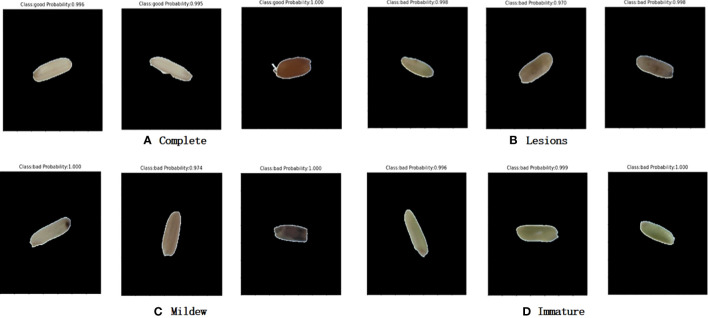
Rice prediction effect. **(A)** Complete. **(B)** Lesions. **(C)** Mildew. **(D)** Immature.

From **Figure 10** and **Table 2**, it can be seen that the training accuracy of the model was 95.5% and the prediction accuracy was 91.3%. The imperfect grains were identified 182 out of 200 sheets, with 91% accuracy; the perfect grains were identified 183 out of 200 sheets, with 91.5% accuracy. The classification accuracy was high. The prediction effect of the model on rice is displayed in **Figure 11**.

#### 4.2.3 Model comparison test

In order to select the optimal model as the classification model of this system, 111 images of perfect grains and 113 images of imperfect grains of the preprocessed rice five-channel images were prepared as prediction sets, which were put into the trained VGG19 network model and Resnet50 network model for prediction. The prediction results were shown in **Table 3**.

**Table 3:** Model prediction effect.

Model	Predict the number of rice grains (Imperfect grain/perfect grain)	Number of imperfect rice grains	Perfect rice grains	Classification accuracy
VGG19	113/111	103	105	92.2%
Resnet50	113/111	101	104	91.3%

It can be seen from the information in the table that the classification accuracy of VGG19 network model is 92.2%, and the classification accuracy of Resnet50 network model is 91.3%. The classification accuracy of VGG19 network model is higher and the training time is less than that of resnet50, which is more in line with the actual production needs. Therefore, VGG19 is selected as the classification model in this system.

### 4.3 Comparison of detection efficiency

#### 4.3.1 Detection time

**Figure 12:**
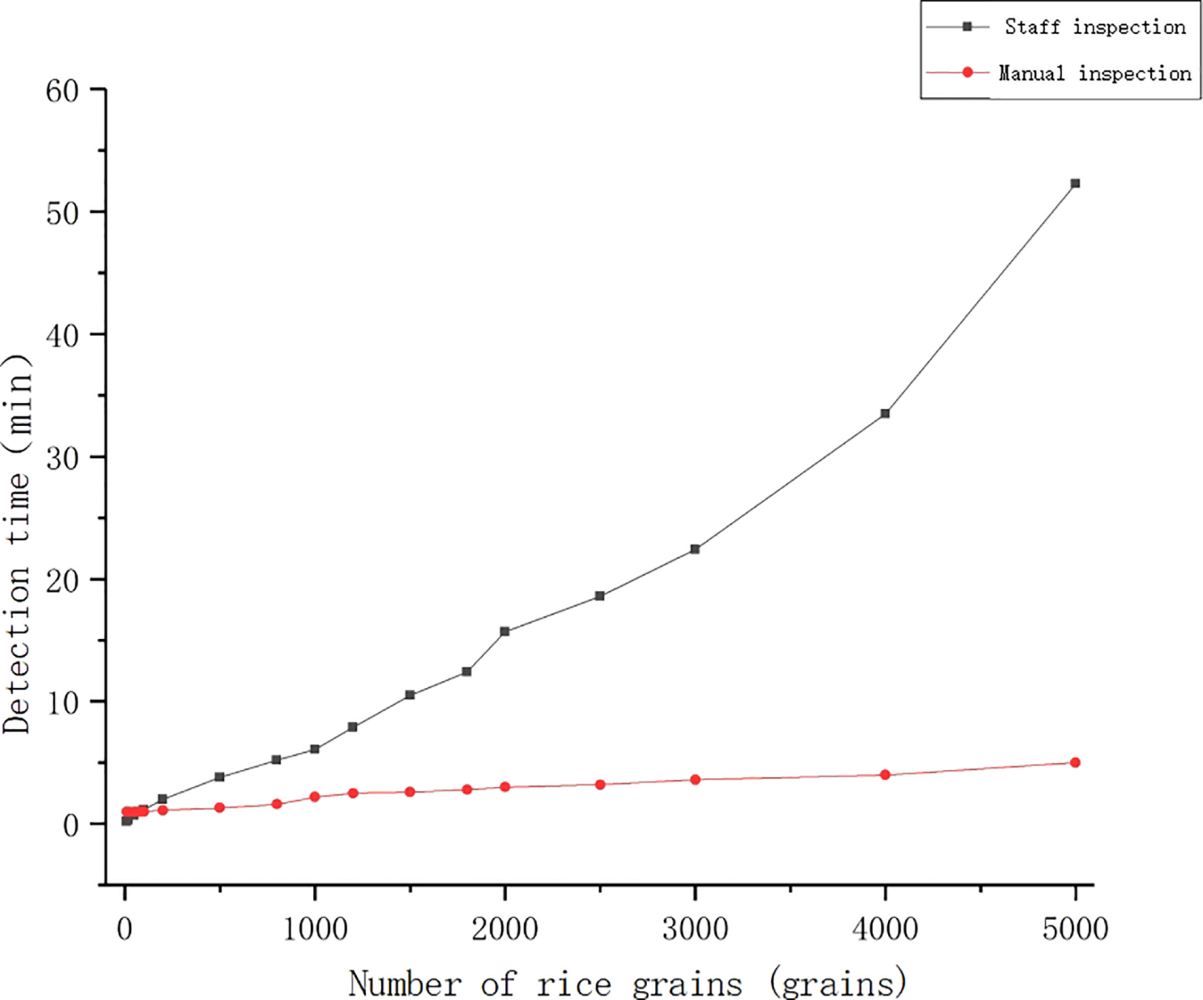
Comparison of detection time.

The experimental data were collected from the National Grain Reserve transfer Warehouse, with manual detection by the national quality inspector for detection and machine detection performed by this research system. The test samples were 12 groups of rice samples including 10, 20, 50, 80, 100, 200, 500, 800, 1000, 1500, 2000, and 5000 grains. The rice was randomly sampled under the same environment, and the corresponding appearance quality tests were performed for different number of grains, the testing time and number of grains were compared and analyzed (see the results of the analysis in **Figure 12**).

As shown in **Figure 12**, the machine inspection did not have an advantage in terms of time when the samples were tested in four groups of 10, 20, 50 and 80 grains, but the machine inspection had a great advantage over the manual inspection for samples above 80 grains. When the test sample was 50 grains, manual test took 0.67 minutes, while machine test took 1 minute. However, when the test sample was 100 grains and 4000 grains, the manual testing time was 1.16 minutes, 33.5 minutes, respectively, and the machine testing time was 1 minute, 5 minutes, respectively. After field research, we knew that most of the rice inspection samples were around 700-800 grains, or even more. Therefore, in practical application, machine test has significant advantages over manual test in terms of testing time.

#### 4.3.2 Detection accuracy

**Figure 13:**
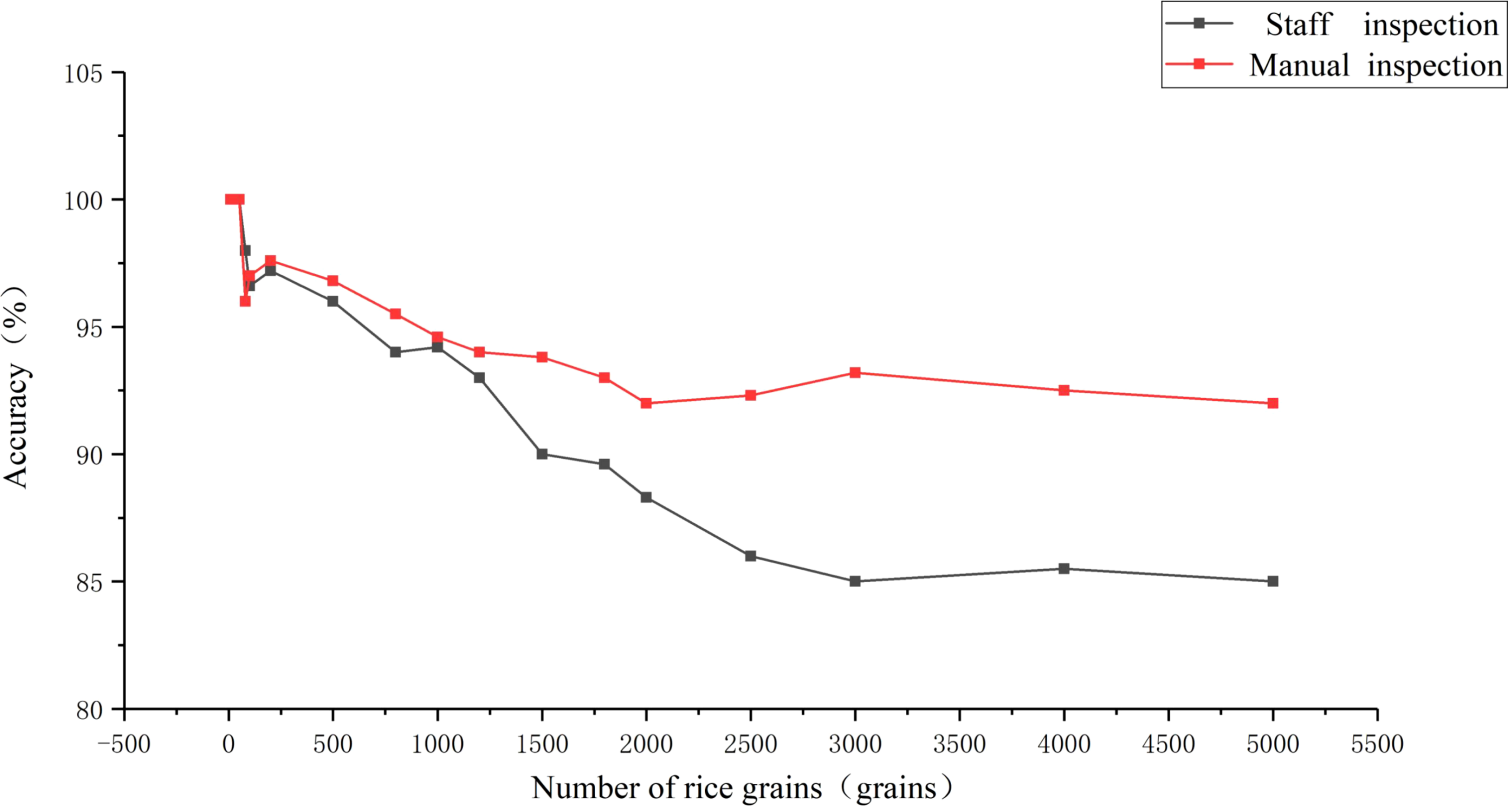
Comparison of measurement accuracy.

The experimental data were collected from the National Grain Reserve transfer Warehouse, with manual detection by the national quality inspector for detection and machine detection performed by this research system. The test samples were 17 groups of rice samples including 10, 20, 50, 80, 100, 200, 500, 800, 1000, 1200, 1500, 1800, 2000, 2500, 3000, 4000, and 5000 grains. The rice was randomly sampled under the same environment, and the corresponding appearance quality tests were carried out for different grain counts, and the accuracy of the tests and the grain counts were compared and analyzed (see the results of the analysis in **Figure 13**).

As displayed in Figure, the machine inspection did not have an advantage in terms of accuracy when the samples were 10, 20, 50 and 80 grains, but the machine inspection had a great advantage over the manual inspection for samples above 80 grains. When the sample was 50, the accuracy of manual detection was 100%, while the accuracy of machine detection was 98.2%. When the samples were 800 and 3000, the accuracy of manual testing was 94.3% and 85%, respectively. While the accuracy of machine testing was 95.5% and 93.2% respectively. As the number of grains tested increased and the testing time grew, the manual test had a fatigue effect and the accuracy rate decreased, while the machine did not have this situation. Therefore, machine inspection has a great advantage over the manual inspection.

## 5 Discussion

In image processing, this study has used Watershed algorithm combined with adaptive thresholding function. Gaussian filtering can effectively remove the noise points in the image binarization process, and the algorithm for calculating pixel point de-impurity is added for the characteristics of binary images to get clear pre-processed images quickly and effectively. The Watershed algorithm alone could produce over-segmentation due to subtle grayscale changes on the surface of the object in the image. In order to avoid the phenomenon of over-segmentation, the adaptive thresholding function is added to avoid the trouble of hand-adjusted thresholding and to eliminate the over-segmentation caused by small changes in grayscale. Both improve the segmentation accuracy and avoid the over-segmentation phenomenon.

Before the establishment of the model, the input data uses five channels of image data. Most of the previous convolutional neural network training used RGB three-channel images, and the accuracy of the trained model was high, but the stability of the model could not be guaranteed. The five-channel image data uses RGB three-channel and two-channel NIR images. The NIR images can capture the smoother seed surface, and the infrared light has a longer wavelength, which is less likely to trigger scattering refraction on the surface and has stronger penetration. Hence, it will not be blocked by the pigment on the epidermis, and the information of the epidermis itself can be better reflected. Compared with the RGB three-channel input image data, the five-channel image reflects more information, and thus the trained model has the higher accuracy and stability.

In the selection of the models, the VGG19 network model has been chosen as the deep learning model of this system in this study. The VGG19 network model and the Resnet50 network model were compared in terms of the accuracy of rice classification. According to the results, the discriminating accuracy of the VGG19 network model was 92.3% and that of the Resnet50 network model was 91.3%. The former is obviously higher than the latter. Thus, the VGG19 network model is chosen as the discriminatory model of this system.

In terms of the detection efficiency, as the manual detection increases with detection time and sample size, the proficiency is improved, but the testing personnel get tired, leading to a decrease in detection efficiency. Therefore, this study compares the detection time and detection accuracy of machine detection and manual detection respectively. As shown by the comparison results, when the detection sample is less than 100 grains, machine detection does not have an advantage over manual detection. However, after the actual research, it was found that the sample size of rice inspection was mostly around 1000 grains, and when the inspection sample was 1000 grains, the time and accuracy of manual detection were 6 min and 94.2% respectively, while the time and accuracy of machine detection were 2 min and 94.6% respectively. That is to say, in practical applications, machine inspection has significant advantages over manual inspection.

## 6 Conclusion

Given the backwardness of the current stage of rice appearance quality inspection level and the emergence of various inspection technologies, this study has proposed a method for rapid automatic inspection of rice appearance quality. With the characteristics of rapid, accurate, convenient and nondestructive detection, it has been significantly improved in automation and intelligence. Based on machine vision technology and image processing technology, the method uses convolutional neural network to establish a strong and stable rice classification model. At the same time, the watershed algorithm and adaptive threshold function are used to segment the image processing as well as the rice grains with good effect, ensuring the accuracy and stability of network training. Apart from that, the VGG19 network model and Resnet50 network model are established. According to the results, the classification accuracy of the model established by the VGG19 network model could reach 92.3%. Thus, VGG19 is selected as the classification model of this system. After experimental verification, the rice appearance quality detection system independently designed in this study has good use experience, has a good alternative to the traditional detection methods, significantly improves the detection efficiency of rice appearance quality, realizes the rapid automatic detection of rice appearance quality. Meanwhile, it positively affects the rice storage and transportation industry. Rice quality inspection technology has always been a popular research direction. With the increase in people’s requirements for rice quality, innovative challenges in quality inspection technology have emerged. The gradual replacement of time-consuming and laborious manual inspection by rice appearance inspection technology has also become a new method in the industry. Therefore, it is essential to continuously develop and discover more new rice appearance quality testing methods so as to improve our rice quality standard system.

## Data availability statement

The original contributions presented in the study are included in the article/supplementary material. Further inquiries can be directed to the corresponding author.

## Author contributions

YH: Writing-original draft. XS: Guiding, Supervision. BF: Data collection. YL: Data process. LS, JZ: Participating in the discussion. XF: Editing, Supervision, Proofreading. All authors contributed to the article and approved the submitted version.
